# Analyzing the relationship between air pollution and various types of crime

**DOI:** 10.1371/journal.pone.0255653

**Published:** 2021-08-13

**Authors:** Pei-Fen Kuo, I. Gede Brawiswa Putra

**Affiliations:** Geomatics Department, National Cheng Kung University, Tainan, Taiwan; National Taiwan University, TAIWAN

## Abstract

Air pollution has a severe impact on human physical and mental health. When the air quality is poor enough to cause respiratory irritation, people tend to stay home and avoid any outdoor activities. In addition, air pollution may cause mental health problems (depression and anxiety) which were associated with high crime risk. Therefore, in this study, it is hypothesized that increasing air pollution level is associated with higher indoor crime rates, but negatively associated with outdoor crime rates because it restricts people’s daily outdoor activities. Three types of crimes were used for this analysis: robbery (outdoor crime), domestic violence (indoor crime), and fraud (cybercrime). The results revealed that the geographically and temporally weighted regression (GTWR) model performed best with lower AIC values. In general, in the higher population areas with more severe air pollution, local authorities should allocate more resources, extra police officers, or more training programs to help them prevent domestic violence, rather than focusing on robbery.

## 1. Introduction

### 1.1 Impact of air pollution

In recent years, the problem of air pollution has become much more serious. According to the World Health Organization’s Global Health Risk report published in 2016, three million people died because of air pollution worldwide. Approximately 87% of these deaths occurred in low and middle-income countries, which represent 82% of the world’s population [1]. The accumulation of air pollutant density and the length of exposure have many negative impacts on human health, including both physical and mental illnesses. However, most researchers have exclusively focused on the former, such as respiratory, cardiovascular, nervous, urinary, and digestive system problems [2,3]. Relatively few scholars have examined the effects of air pollution on mental health and how it could lead to changes in human behavior and daily activity. For example, Shin *et al*.,[4] and Zeng et al [5] argue that air pollution could increase stress and inflammatory reactions. They have found an association between the subjective stress of daily activities and long-term exposure to ambient air pollutants. According to Shin *et al*. [4], men as well anyone under the age of 65 are at greater risk of developing mental problems because these groups are more likely to have been exposed to air pollution due to their higher mobility rates and greater activity levels. Lu et al [6], who explore the correlation between air pollution’s effect on the mental health and increases in crime rates, found that severe air pollution, increases the anxiety levels of the respondents and may lead people to behave unethically. The reason is the anxiety might arise in threatening situations, and then the brain will develop self-protection responses [7,8], which make people focus more on their own personal needs and self-interests, and therefore they become less mindful of the ethical morals principle that causing them to act unethically [8]. Therefore, since air pollution can directly affect the human body and mind, it may indirectly associated with human behavior and crime rates.

Recently, several scholars have performed large scale observational studies using open datasets to analyze the relationships among air pollution and various crimes [9–11]. Among various types of crime, the association between air quality and crime varies for property and violent crimes. Herrnstadt et al. [10] found an increase of 6.14 percent in violent crime when air quality deteriorated due to the occurrences of windy days that brought dirty air in Los Angeles. Similarly, their study case in Chicago also had 2.2 percent increase in violent crime when the downside wind came. This effect was expected to occur on the same day where the high concentration of air pollution on that day only impacted the number of criminals on the same day [11]. The authors speculated that excessive concentrations of air pollution could lead to more aggressive behaviour and, hence, violent crime increased. On the other hand, Baryshnikova [12] found a negative correlation between air pollution and property crime. A ten percent increase in ozone concentrations reduced the number of crimes committed hourly by 0.68 percent. As air pollution levels increased, people avoided leaving their homes, and this reduced the likelihood of a successful property crime. Although scholars have determined that air pollution is associated with crime, they have only begun to explore its influence in a large-scale study area and have not specified how they are correlated locally.

### 1.2 Crime, activity level, and air pollution

There are many complex factors which are correlated with crime, such as opportunity, location, the behavior of both the offender and the victim, and time [13]. According to the routine activity theory, people’s daily comings and goings may determine where and when a vulnerable victim and a motivated offender might come into contact with each other. For example, robberies usually take place in public areas such as restaurants, on public transportation, in parking lots, and in banks [14], whereas domestic abuse usually occurs at home. According to Khan [15], there are two types of crime scenes: indoor crimes and outdoor crimes. Therefore, changes in people’s lifestyles and daily activities can provide opportunities for potential perpetrators and related with the spatial and temporal distribution of crime. Tompson and Bowers [16] applied the negative impact escape (NAE) model to an early study conducted by Rotton and Cohn [17] to determine if bad weather is associated with crime risk. According to the model, there is a steady linear temperature-violence relationship in which extreme temperatures (too hot or too cold) may be associated with aggressive behavior and violent crimes.

According to Yan *et al*. [18], an increase in air pollution affects how people get to work and leads to a decrease in the number of people who use public transportation to reduce exposure risk from air pollution by using private vehicle and avoiding waiting time for the public transit. In addition, Evans [19] found that when pollution is severe, people limit their outdoor leisure activities but do not change their indoor activities and tend to spend more time indoors. Taking Moretti and Neidell’s [20] study as an example, when air pollution levels became dangerous, the number of visitors to the Los Angeles Zoo drops significantly. Thus, high levels of air pollution change people’s activity patterns because they are unwilling to go out and subject themselves to possible health risks.

### 1.3 Socio-economic factors

In terms of outdoor crime, Monk *et al*. [21] argue that offenders, location, and victim’s routines all contribute to street robbery. The motivations for this type of crime are lack of income, unemployment, and drug use in the area [22]. Some offenders appear to have a plan, while others strike randomly. For example, potential robbers may look for victims who are vulnerable, particularly those who lack awareness of their surroundings. According to Wolff and Asche [14], a high number of pedestrians is associated with an increase in the number of robberies. Also, as previously stated, location and routine create an opportunity for robbers, and typical settings are on public transportation, banks, restaurants, liquor stores, and bars [21,23].

Several studies from around the world clearly show that domestic violence is a significant public health problem [24]. Most victims of this typically indoor crime are women and children. Moreover, forty-nine percent (49%) of women have been assaulted by a male under the influence of alcohol or drugs [25]. Hence, the related factors include the male-to-female ratio, economic conditions [26], and drug use. Also, victims who have experienced child abused before the age of 15 have a higher risk of becoming perpetrators of domestic violence themselves. Although according to this personal dataset, violence experienced in childhood and family background play a critical role in domestic violence, it is difficult to access and is beyond the scope of this study. According to previous studies [27–30], air pollution may be directly (cognitive function) and indirectly (physical health, and behaviour) be associated with mental illnesses including depression and anxiety. There is also an evidence that the latter may contribute to both violence and aggression [9,31]. For instance, anxiety due to negative social changes (for example, economic distress) may cause individuals to become more aggressive and hostile because if people stay at home for extended periods, they are likely to become more stressed, which may increase negative attitutes toward family members [32]. This stress can then lead to high rates of domestic violence. The literature provides significant evidence of psychological stress associated with domestic violence which triggers new instances of partner abuse [33].

Online fraud is a widespread phenomenon, not just in terms of its economic impact on the government and businesses but also in terms of its harmful effects on many individuals. According to the Australian Payments Network [34], online fraud accounted for approximately 0.03 percent of the total transaction using cards and cheques in 2016 ($540 million). If the routine activity theory is applied to online fraud violations, it becomes obvious that there are millions of potential victims to be targeted anywhere in the world via the internet today [35]. According to this theory, people who spend long periods of time on the internet put themselves at greater risk of falling victim to online fraud [36].

### 1.4 Contributions of this study

Historically, relatively few scholars have studied the relationship between air pollution and crime [9,10,37–39]. However, currently there are several researchers who have shown an interest in this topic and have analyzed this correlation using global models to illustrate this association in general [11,40,41]. Surprisingly, there has been very little detailed discussion of how air pollution correlates to mental health and increases crime risk, or how changes in behavior and activities are evaluated with regard to the rates of certain types of crime.

Unlike most previous research, this study examined the relationship between the air quality index on both indoor and outdoor crimes. Based on the literature review results and the limitations of the data, in this study, the air pollution index and other nine weather and socio-economic variables (such as population, male-to-female ratio, income, unemployment rate, divorce rate, ages) were chosen to build crime prediction models. In the pilot study [42], a shorter air quality monitoring period and fewer weather stations were used to predict the relation of air pollution to robbery and domestic violence. The results showed that geographically weighted regression (GWR) performed better than OLS. However, the GWR model only focused on the interaction between spatial units while ignoring the relationship between temporal units. In this current study, the geographically and temporally weighted regression (GTWR) model was used to monitor the dynamic changes over time as well as the spatial dependence of variables. In addition, this method was compared to the previous model utilized in existing studies to illustrate the impact of the temporal effect on GTWR. Also, the model results could be used to predict the spatial-temporal hotspots of crime and then to develop effective crime prevention policies for each district in different seasons.

## 2. Study data and methodology

### 2.1 Study area and data

**Fig 1:**
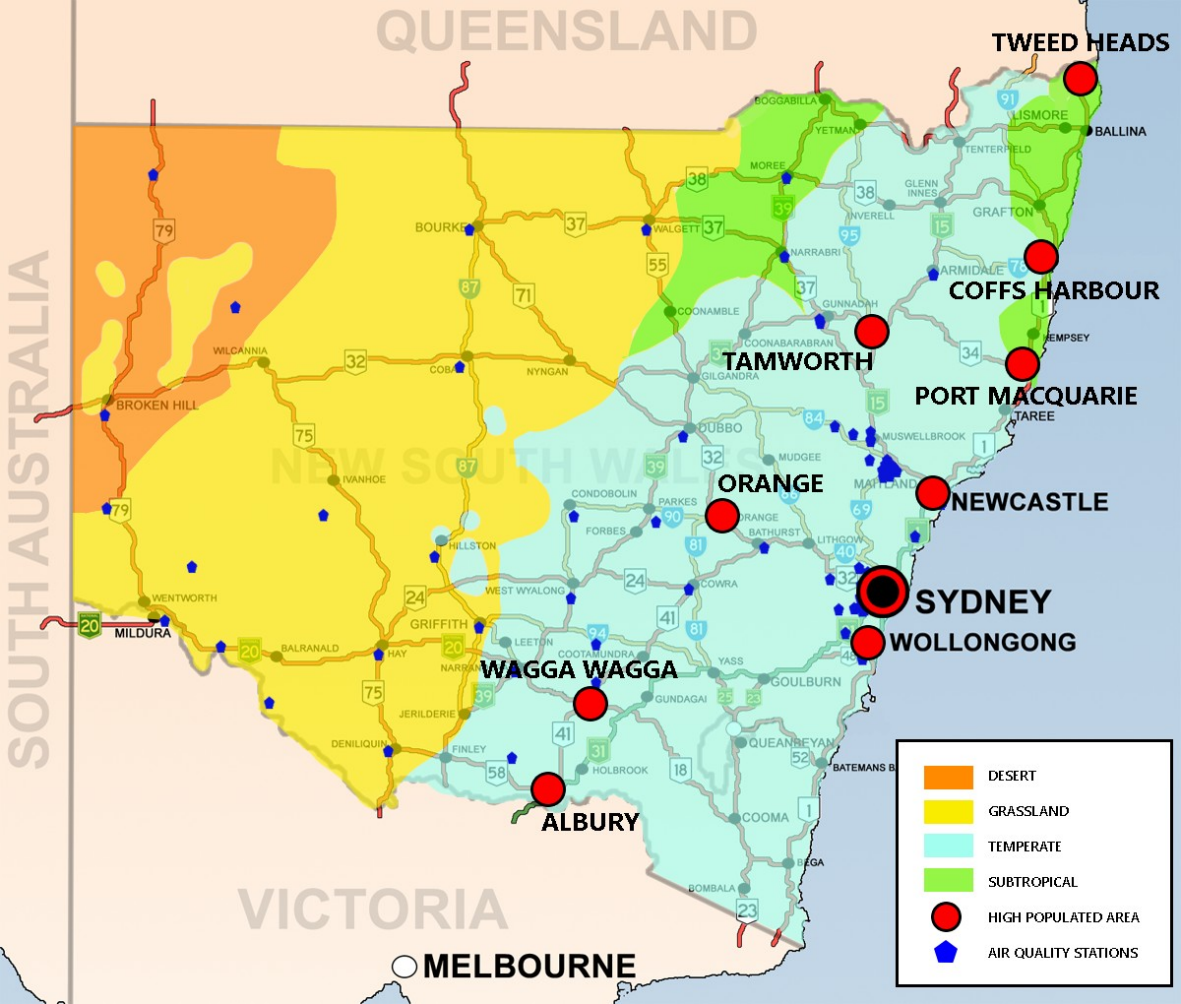
Study area map.

The study area of New South Wales, Australia (NSW) had a population of over eight million as of 2019 and has become the most populous state on the continent. The majority of these people (5.1 million) live in the Greater Sydney Area, the capital city of New South Wales, which is Australia’s most famous business and tourist city and the most densely populated. This density of population and constant activity in the cities of NSW is associated with a high crime rate and worsening air pollution. The units included in this study were 129 Local Government Areas (LGA). The red points in Fig 1 denote major cities, including Sydney, Newcastle, Wollongong, Albury, Tweed Heads, and others. Most of the western areas are desert and grassland, which have low population density and minimal crime risk. Therefore, the following analysis focused on the coastal areas that have higher concentrations of air pollution and higher crime risk.

**Fig 2:**
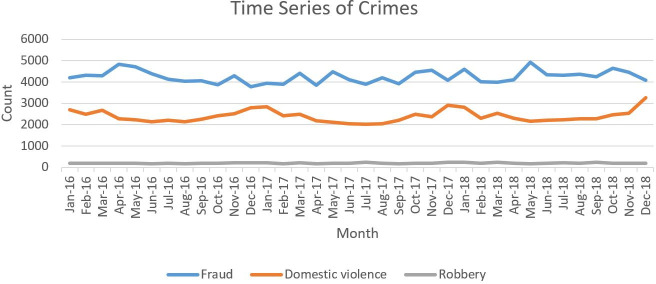
Time series of crime in New South Wales.

This study focused on three types of crime that occurred in New South Wales in 2016 until 2018: domestic violence, robbery, and fraud. According to the Bureau of Crime Statistics and Research of NSW, domestic violence is defined as “an offense or abuse against a person in the form of assault, harassment, or another similar behavior.” Robbery is described as “taking property or money from a person through force or by fear of force.” Fraud is “a course of action, by deceit or other dishonest conduct, with the intent to obtain money or other benefits or to evade liability.” The crime datasets are posted every month on the 129 LGA, which can be downloaded from the Bureau of Crime Statistics and Research’s official website (www.bocsar.nsw.gov.au). Fig 2 illustrates the crime trends in 2016 until 2018, which shows that instances of fraud were the most frequent at 4,000 cases each month followed by domestic violence and robbery.

### 2.2 Air pollution and weather data

*The air quality index (AQI) is utilized for reporting daily air quality*. *The daily pollutant measurements include ozone*, *nitrogen dioxide*, *carbon monoxide*, *sulphur dioxide*, *and particulate matter (PM2*.*5 and PM10)*. *The dataset was downloaded in 2016 until 2018 from 87 air quality stations from the Department of Planning*, *Industry*, *and Environment’s official website in NSW (**www*.*dpie*.*nsw*.*gov*.*au**). These stations are located in almost every part of the study area except the south-east corner (denoted by the blue point in Fig 1). To determine the daily AQI value, each contaminant index is calculated at the station, and the highest is selected as the daily value. The calculation formula to determine the AQI value is based on the New South Wales standard, as shown in* Eq *(1)*.

$${AQI}_{i,j}=max(\frac{p_{i,j}}{ps}\times 100)$$ (1)

where p_*i,j*_ is a concentration of pollutants on the *i*-th day at location *j*; ps is a standard concentration of pollutants as designated by the Department of Planning, Industry and Environment in New South Wales, shown in Table 1; and *AQI_i,j_* is the AQI level on the *i*-th day at location *j*, which is the maximum values of the division of each pollutant’s concentration at the day to its corresponding standard and status (Table 2).

**Table 1:** New South Wales pollutant standard concentration.

Pollutant	Averaging period	Air NEPM Standard (ps)	NSW reporting format
Carbon Monoxide	8 hours	9.0 ppm	9.0 ppm
Nitrogen Dioxide	1 hour	0.12 ppm	12.0 pphm
Ozone	1 hour	0.10 ppm	10.0 pphm
	4 hours	0.08 ppm	8.0 pphm
Sulfur Dioxide	1 hour	0.20 ppm	20 pphm
PM10	1 day	50 μg/m3	50 μg/m3
PM2.5	1 day	25 μg/m3	25 μg/m3

Source: Department of Planning, Industry, and Environment in New South Wales.

**Table 2:** New South Wales AQI Standard Status.

AQI Value	Status	Recommendation
0–33	Very good	Usual activities
34–66	Good	Usual activities
67–99	Fair	People who are exceptionally susceptible to air pollution need to postpone their outdoor activities
100–149	Poor	**Sensitive Groups:** Cut back or reschedule strenuous outdoor activities
150–200	Very poor	**Sensitive groups:** Avoid strenuous outdoor activities
		**Everyone**: Cut back or reschedule strenuous outdoor activities
200+	Hazardous	**Sensitive groups:** Avoid all outdoor physical activities
		**Everyone:** Significantly cut back on outdoor physical activities

Source: Department of Planning, Industry, and Environment in New South Wales.

As the crime data is only available monthly, this study therefore, also used monthly AQI data based on the several days’ observations in each month. Although the monthly AQI values were not categorized as a hazardous level, there were several days of observation that exceeded the national air pollution standard. In Sydney, the particulates concentrations that surpassed the national standards were up to 40 days a year and up to 30 days a year in the rural areas during the study period.

Monthly weather data, such as temperatures and rainfall, were collected for each area and provided by its local meteorological agency (http://www.bom.gov.au/). In this study, maximum and minimum temperature conditions were collected at each monitoring station. It must be noted that rainfall includes all forms of precipitation that reach the ground, including rain, drizzle, hail and snow.

**Fig 3:**
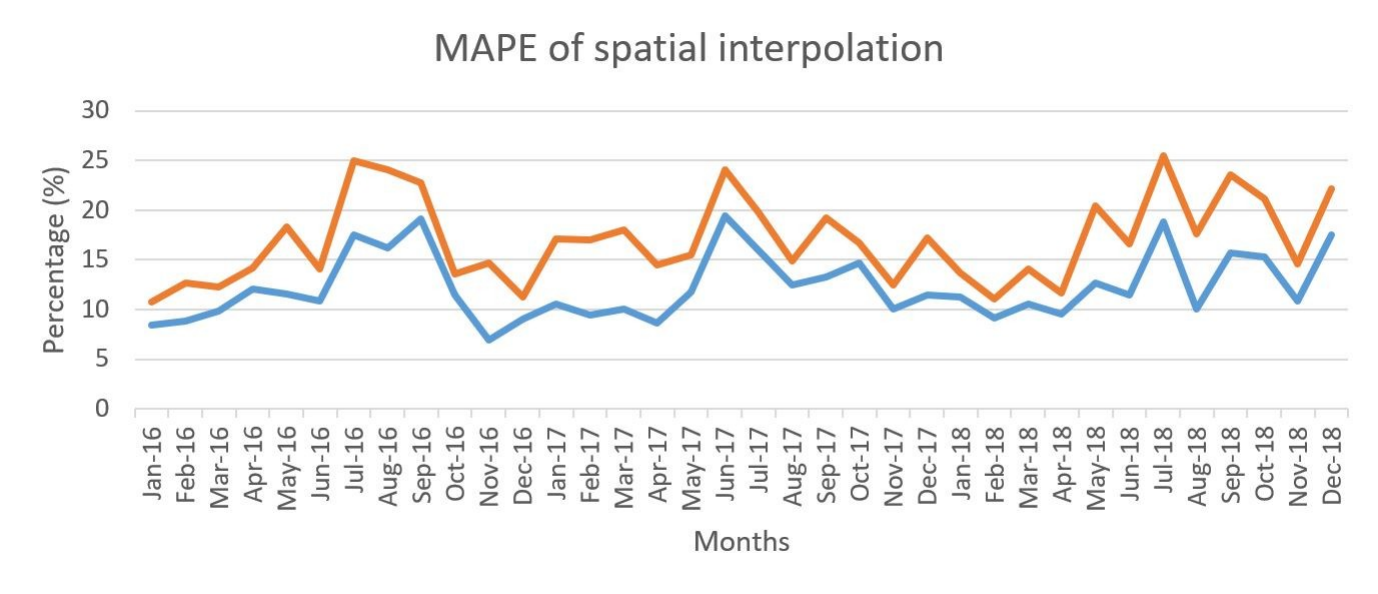
Cross-validation results of ordinary kriging and IDW for AQI in New South Wales.

Due to the limited number or complete lack of air quality and weather monitoring stations, the spatial interpolation method was used to estimate weather condition. (Please see Fig 1 for the locations of air quality monitoring stations.) The two most popular methods, inverse distance weighting (IDW) and kriging, were selected to fill in the missing data values. The IDW method is relatively straight-forward to compute. In this study, we set 12 nearby stations as the search radius for interpolation using a 10-meter output cell size. However, IDW is not the most suitable method for determining the spatial variations of relationships between points, so the accuracy of the estimation is limited [43]. In comparison, to estimate the arrangement of spatial variance, we also used the Kriging approach, which utilizes variogram analysis and takes into account spatial autocorrelation [44,45]. Among all kriging methods, we chose the ordinary kriging with a spherical semi variogram model. The search radius and the output cell size were the same as those selected for use with the IDW method. According to the mean absolute percentage error (MAPE) values from cross-validation, the ordinary kriging method consistently performed better than IDW (Fig 3), which was confirmed by a smaller MAPE value that indicates less of a difference between the observed and predicted values. Then, the results of ordinary kriging were used in this study as the independent variables of AQI.

### 2.3 Social economic variables

In addition to the AQI and weather variables, other socio-economic factors may directly or indirectly associated with crime rates (please see Appendix A for the correlation table) [46–49]. Based on previous studies [11,42], in order to improve the model’s predictive power and to control for external variables, for this study population, the male-to-female ratio, median age, education, income, divorce rate, and unemployment rate of cities in the crime prediction model were included. Table 3 shows the descriptive statistics of these variables in NSW, which were downloaded from the Australia Bureau of Statistics (*www*.*abs*.*gov*.*au*).

**Table 3:** Descriptive statistics of the variable.

Variables	Mean	Min	Median	Max
Domestic Violence (log)	0.899	0	0.903	2.386
Robbery (log)	0.223	0	0	1.613
Fraud (log)	1.024	0	0.954	2.769
Total Population (1000 unit)	57.892	1.056	22.987	34.630
Female Percentage(%)	50.33	46.3	49.5	59
Median Age	41.23	36	42	54
Level of Education (%)	17.04	6.01	11.5	43.5
Weekly Income (AUD)	667.838	478	611.033	1454
Unemployment (%)	6.116	1.3	5.9	16.2
Divorce (%)	8.879	4.9	8.6	12.5
Average Observed Rainfall (mm)	57.940	0	38	806.8
Minimal Temperature (^o^C)	11.506	-4.5	11.8	25.5
AQI (index)	40.586	10.8	39.2	114.7

Sample size: 4644 including, 129 LGA; 36 months observation.

### 2.4 Methodology

The introduction of the GWR model is necessary before GTWR is used. The former is commonly utilized for investigating the influence of the predict variables on independent variable because it allows spatial variable parameters. With the spatial data of air pollution and crime, an analysis of the association between air pollution and crime could be illustrated in more detail. Moreover, local models can be made that could show the correlation of air pollution in each location i. Its fundamental formula is described as follows [50]:

$$Y_{1i}=\beta_{0}(u_{i},v_{i})+\beta_{k}(u_{i},v_{i})X_{ik}+\varepsilon_{i}$$ (2)

Where, Y_1i_ is the number of domestic violence crimes (first type) committed in each location of the study area. The second type is robbery and the third is fraud; the X_ik_ independent variable includes socio-economic factors, weather, and air quality index values (AQI); (u_i_, v_i_) is the geographical coordinates of the LGA_i_; β_k_ (u_i_, v_i_) is the k^th^ parameter of the LGAi^th^; ε_i_ is the random error of the i^th^ sample. Unlike other models, parameters βk(ui, νi) in the GWR are allowed to vary across the model to more effectively measure the spatial non-stationarity of observations compared to the global model in which parameter estimation is fixed for each observation.

However, GWR cannot handle temporal non-stationarity effects, which is another critical influential factor in this study. For parameter estimates, the GWR model can be used to account for spatial non-stationarity by establishing a weight matrix according to the distances between point i and all the other observations. It is common to adjust the time variables individually to avoid spurious regression that occurs as a result of temporal non-statonarity by only selecting one-day data or aggregating the number of event observations to a specific time period. However, due to the fact that our study data on air quality is usually classified as temporally non-stationary, this time variable cannot be ignored.

Therefore, the Augmented Dickey-Fuller test (ADF) was used to identify whether a given time series data was stationary or not. It was chosen because it is more effective and robust than other staionarity assessments, such the Phillips-Perron (PP), as well as the original Dickey-Fuller (DF) test. The null hypothesis of the ADF test stated that there is a unit root, meaning that the data is non-stationary, while the alternative is that there is no unit root [51]. For example, a unit root exists in a time series when the value of alpha is equal to one, as shown in Eq 3 below:

$${\Delta X}_{ik,t}=\alpha X_{ik,t-1}+\beta_{1}X_{ik,t-1}+\ldots +{\beta_{p}X}_{ik,t-p}+\varepsilon$$ (3)

where, X_ik_,_t_ is the value of the time series variable in location i for every single k variable, at time t, and *p* denotes the number of lags. In this test, if the P-value is less than 0.05, this indicates that the data sequence is stationary. Thus, the null hypothesis must be rejected. However, if the P-value is higher than 0.05, the data pattern will be non-stationary [51,52]. If the null hypothesis of ADF test is not rejected and the time series data is non-stationary, the GTWR model (proposed by Huang et al. [53]) will be more accurate than the GWR because it is able to integrate both spatial and temporal variables into the weight matrices in order to capture temporal and spatial heterogeneity.

This study utilized the GTWR method to account for the spatial-temporal non-stationary parameter estimates as an alternative strategy by building the weight matrix based on the distances calculated from the (u, v, t) coordinates between observation i and all the other observations. Thus, the GTWR model can be expressed in Eq (4).


$$Y_{it}=\beta_{0}(u_{i},v_{i},t_{i})+\sum_{k}\beta_{k}(u_{i},v_{i},t_{i})X_{ik}+\varepsilon_{i}$$ (4)

The main challenge is to provide estimates of β_k_ (u_i_,v_i_,t_i_), for every single k variable and each space-time location i. In this way, the estimation of β_k_ (u_i_,v_i_,t_i_) can be expressed in Eq (5):

$$\hat{\beta }(u_{i},v_{i},t_{i})=[X^{T}W(u_{i},v_{i},t_{i})X{]}^{-1}X^{T}W(u_{i},v_{i},t_{i})Y$$ (5)

Where, W(u_i_,v_i_,t_i_) is a diagonal matrix (α_i1_, α_i2_,…, α_in_); α_ij_ (1 ≤ j ≤ n) denotes the spatial-time distance functions of u,v,t that correspond to weights when the weighted regression of the observation LGA_i_ is calibrated; X represents observations of the independent variables, including urban areas, population density, male-to-female ratio, median age, income, percentage of the population with education levels above a bachelor’s degree, unemployment rate, and monthly air quality index (AQI); and N is the number of observations.


$$d^{ST}={\lambda d}^{S}+\mu d^{T}$$ (6)

Given a spatial distance *d*^*S*^ and a temporal distance *d*^*T*^, those variable could combined to form a spatio-temporal distance *d*^*ST*^. Location distance and time are usually measured in different units (in this case, location in meters and time in months). Thus, they have different scale effects where λ represents the spatial scale and μ is the temporal scale. Therefore, if we let τ denote the parameter ratio μ/λ and λ≠0 [53,54], then, Eq (6) can be rewritten by normalizing the coefficient of d^S^ as follows:

$$\frac{{\left(d_{ij}^{ST}\right)}^{2}}{\lambda }=\left[{\left(u_{i}-u_{j}\right)}^{2}+{\left(v_{i}-v_{j}\right)}^{2}\right]+{\tau (t_{i}-t_{j})}^{2}$$ (7)

$$W_{ij}=exp\frac{{\left(d_{ij}^{ST}\right)}^{2}/\lambda }{{\left(h_{ST}\right)}^{2}/\lambda }$$ (8)

Due to the fact that W(u_i_,v_i_,t_i_) is multiplied by a constant value, it will not influence the estimation of β_k_(u_i_,v_i_,t_i_). Thus, the parameter ratio τ = μ/λ plays an essential role in constructing the weights matrix. In fact, the main purpose of τ is to increase/reduce the temporal distance effect to match the spatial distance.

With no loss of generality, when the spatial scale is set to λ = 1 to reduce the number of parameters in play, only μ must be determined and can be optimized using cross-validation in terms of R-square or AIC if a priori knowledge is not available.

The selection of the bandwidth h_S_ is another important step for calibrating the model. The cross-validation (CV) method can be used to select this parameter. If we assume that y_≠i_(h_S_) is the expected value yielded from the GTWR model with a bandwidth of hS, the sum of the squared error could be written as follows:

$$CV(h)=\sum_{i}{\left[y_{i}-y_{\neq i}(h_{s})\right]}^{2}$$ (9)

In practice, fluctuations in the sum of the squared error can be observed by plotting CV(h) against parameter h to select the optimal parameter h. Also, minimizing CV(h) in terms of goodness-of-fit statistics or the corrected Akaike information criterion (AIC) is another effective technique for selecting parameter h [54].

## 3. Results

### 3.1 Crime prediction model fitting results

In order to illustrate how the association of the predictor variables and crime changes over location and time, three different models (two GWR-based models and OLS) were built and their performance was then compared. It must be noted that the non-stationary variables could cause spurious regression results that lead to a false statistical significance, thus rendering the OLS model unreliable [52,55]. Therefore, the ADF test was implemented to determine if the weather variables were temporally stationary. If these test results reject the null hypothesis, the data will be identified as stationary [51,52]. Table 4 shows the results of the ADF test (including the ADF statistics and a 95% confidence rate). The high proportion of negative ADF statistics indicated a stronger stationary data pattern at certain confidence levels. On the other hand, the 95% confidence rate showed that the ratio number of LGA accepted the null hypothesis at p-value 0.05. For example, according to the ADF test of the AQI, 92.2% of New South Wales showed a p-value greater than 0.05, and the AQI ranged from -3.89 to -2.35. Since 92.2% of the area accepted the null hypothesis, the AQI variable was shown to be non-stationary. Another example is the temperature variable which ranged from -5.18 to -3.27. In contrast to the AQI variable, there was only a 10.8% area that accepted the null hypothesis. Thus, this data indicated that the temperature variable was temporally stationary. However, the ADF test on rainfall showed that 75.2% of the area had a p-value below 0.05, which indicated that this variable was temporally non-stationary. Therefore, the GTWR model was used based on these ADF results.

**Table 4:** The results of the ADF test for variable sequences.

Variables	ADF Statistics			95% confidence rate	Results
	Min	Median	Max		
Temperature	-5.18 *	-3.83	-3.27	10.8%	Stationary
Rainfall	-4.23	-3.15	-2.93	75.2%	Nonstationary
AQI	-3.89	-2.97	-2.53	92.2%	Nonstationary

Significant Code: 0.0001’***’; 0.001’**’; 0.01’*’; 0.05 ‘.’; 0.1 ‘ ‘.

Furthermore, to test the accuracy of the model estimates, the spatial non-stationary data can be assessed by comparing twice the standard errors of the global ordinary least squares (OLS) estimators to the interquartile (i.e., the differences between the lower and upper quartiles) of the local estimators of GWR and GTWR. For the latter, the lowest interquartile of cross sectional GWR coefficient value was run for 36 months in order to compare it to the OLS estimator. The larger the interquartile value, the more significant the spatial non-stationarity. Then, the local model should be implemented to examine these spatio-temporal effects [50,56]. According to Table 5, most socio-economic and AQI variables have higher interquartile values more than twice the OLS standard errors (with the exception of the income variable), such as population, female ratio, median age, level of education, divorce rate, unemployment rate, rainfall, and temperature variables, indicating that the coefficient values were spatial-temporally heterogenous. However, the differences between the interquartile and the standard error values for income were relatively minimal, especially for robbery crime. This is because this coefficient has minimal variance and this variable did not differ greatly in neighboring locations.

**Table 5:** Spatial non-stationary tests of variables.

Variable	Domestic Violence			Robbery			Fraud		
	Interquartile (GTWR)	Lowest Interquartile (GWR)	2 x SE (OLS)	Interquartile (GTWR)	Lowest Interquartile (GWR)	2 x SE (OLS)	Interquartile (GTWR)	Lowest Interquartile (GWR)	2 x SE (OLS)
Population	0.031	0.021	0.013	0.003	0.002	0.001	0.077	0.035	0.027
Percentage of Female	0.588	0.382	0.232	0.033	0.024	0.019	1.410	0.567	0.502
Median Age	0.570	0.396	0.220	0.039	0.022	0.018	1.020	0.551	0.475
Income	0.012	0.010	0.010	0.001	0.001	0.001	0.018	0.016	0.016
Percentage of Divorce	1.420	0.883	0.571	0.153	0.055	0.047	2.860	1.590	1.234
Percentage of those with Higher Education	0.349	0.268	0.143	0.031	0.020	0.012	0.672	0.495	0.310
Unemployment Percentage	0.764	0.551	0.307	0.080	0.033	0.026	1.740	0.727	0.663
Min Temperature	0.385	0.211	0.147	0.029	0.011	0.012	0.703	0.323	0.318
Rainfall	0.026	0.017	0.012	0.002	0.002	0.001	0.060	0.032	0.026
AQI	0.168	0.132	0.081	0.016	0.012	0.007	0.395	0.292	0.174

This study also found that the residuals of OLS model were not normally distributed, and further assesment was also conducted by analyzing the spatial autocorrelation of the OLS residuals using Moran’s I. The results showed that the residuals were significantly clustered. After considering the benefits of the local model, therefore, the GTWR model was utilized to address athe spatio-temporal influence.

Table 6 shows the coefficient estimators and the individual performances of three different models. The performance of the model fitting was evaluated by the R-square and AIC values. The GTWR model was chosen because it had the lowest Akaike Information Criterion (AIC) and the highest R-square value. The AIC value of this model for domestic violence was 2362.8 (AIC for the OLS model was 3198.44); the robbery model was 1807.47 (AIC for the OLS model was 1833.72), and fraud was 2635.69 (the AIC for the OLS model was 3458.02). In addition, the variance inflation factor (VIF) value was used to measure collinearity among variables. When predict variables have a VIF value larger than five, some of them should be removed from the model to avoid this issue [57]. According to the OLS model, all the VIF value was below five, which means that the explanatory variables were not dependent and did not profoundly affect each other.

**Table 6:** Comparison of global and local model results.

Variable	Domestic Violence			Robbery			Fraud		
	OLS	GWR Mean	GTWR Mean	OLS	GWR Mean	GTWR Mean	OLS	GWR Mean	GTWR Mean
Intercept	-0.5842***	-1.8761	-1.8504	0.6559***	0.8166	0.7615	-1.1920***	-1.5524	-1.4861
Population	0.0013***	0.0029	0.0027	0.0012***	0.0023	0.0024	0.0015***	0.0068	0.0073
Female Percentage	0.0251***	0.0229	0.0288	-0.0112	-0.0201	-0.0193	0.0313	0.0032	0.0267
Median Age	-0.0010**	-0.0064	-0.0098	-0.0064**	-0.0089	-0.0085	-0.0026*	-0.0030	-0.0031
Income	-0.0003***	-0.0003	-0.0004	-0.0003***	-0.0013	-0.0015	-0.0003	-0.0009	0.0010
Divorce Percentage	0.0054*	0.0298	0.0238	0.0062	0.0089	0.0085	0.0138*	0.0247	0.0244
Higher Education Percentage	-0.0091***	-0.0081	-0.0086	0.0028***	0.0099	0.0104	0.0167***	0.0124	-0.0125
Unemployment Percentage	0.0329***	0.0894	0.0993	0.0009***	0.0017	0.0015	0.0325***	0.0021	0.0021
Min Temperature	0.0035*	0.0051	0.0070	-0.0002*	-0.0002	-0.0001	0.0070***	0.0021	0.0024
Rainfall	0.0036*	0.0029	0.0022	-0.0006	-0.0002	-0.0002	0.0007*	0.0003	0.0002
AQI	0.0179***	0.0102	0.0145	-0.0012**	-0.0042	-0.0040	0.0056***	0.0074	0.0071
R2	0.539	0.761	0.783	0.389	0.566	0.572	0.606	0.868	0.874
AIC	7301.673	6205.298	6036.690	1965.704	1110.110	1191.480	9447.217	8229.970	8075.010

Significant Code: 0.0001’***’; 0.001’**’; 0.01’*’; 0.05 ‘.’; 0.1 ‘ ‘.

### 3.2 Comparison of the GTWR and GWR models

The GWR model extends the linear regression framework by estimating local parameters via optimal bandwidth. However, since complex temporal effects can also lead to non-stationarity in crime distribution, the GTWR model can capture both spatial and temporal heterogeneity to improve its goodness-of-fit. Huang et al, [53] introduced parameter τ to balance and harmonize various spatial and temporal units in order to calculate the space-time distance before constructing spatio-temporal weighting matrices. Therefore, τ must be optimized before the GTWR can be implemented.

**Fig 4:**
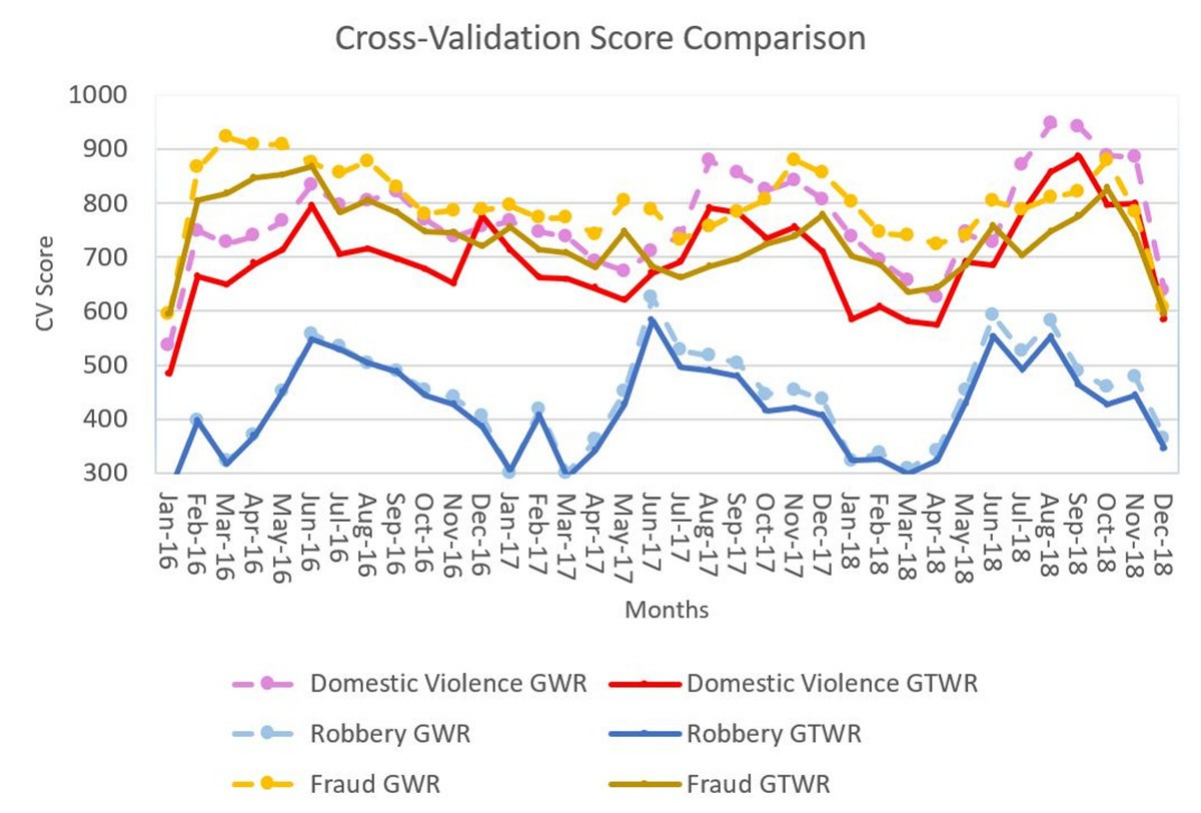
Cross-validation score from the GTWR and cross-sectional GWR results for various types of crime.

Thus, it is essential to compare the model performance of GTWR against the cross-sectional GWR approach when local parameters are estimated each year via GWR by exclusively using the data from the year in which the model was calibrated. Fig 4 shows the annual CV scores of the three crime prediction models, which were calibrated by the cross-sectional GWR and GTWR.

According to Fig 4, the cross-validation scores of GTWR model are consistently lower than GWR model for both domestic violence and fraud. However, the scores in robbery case from both methods at first appear to be similar. Even so, when viewed as a whole, the goodness-of-fit GTWR model was slightly more accurate than the GWR for all the months of the study period, which is consistent with previous studies [53,54,56,58,59].

**Fig 5:**
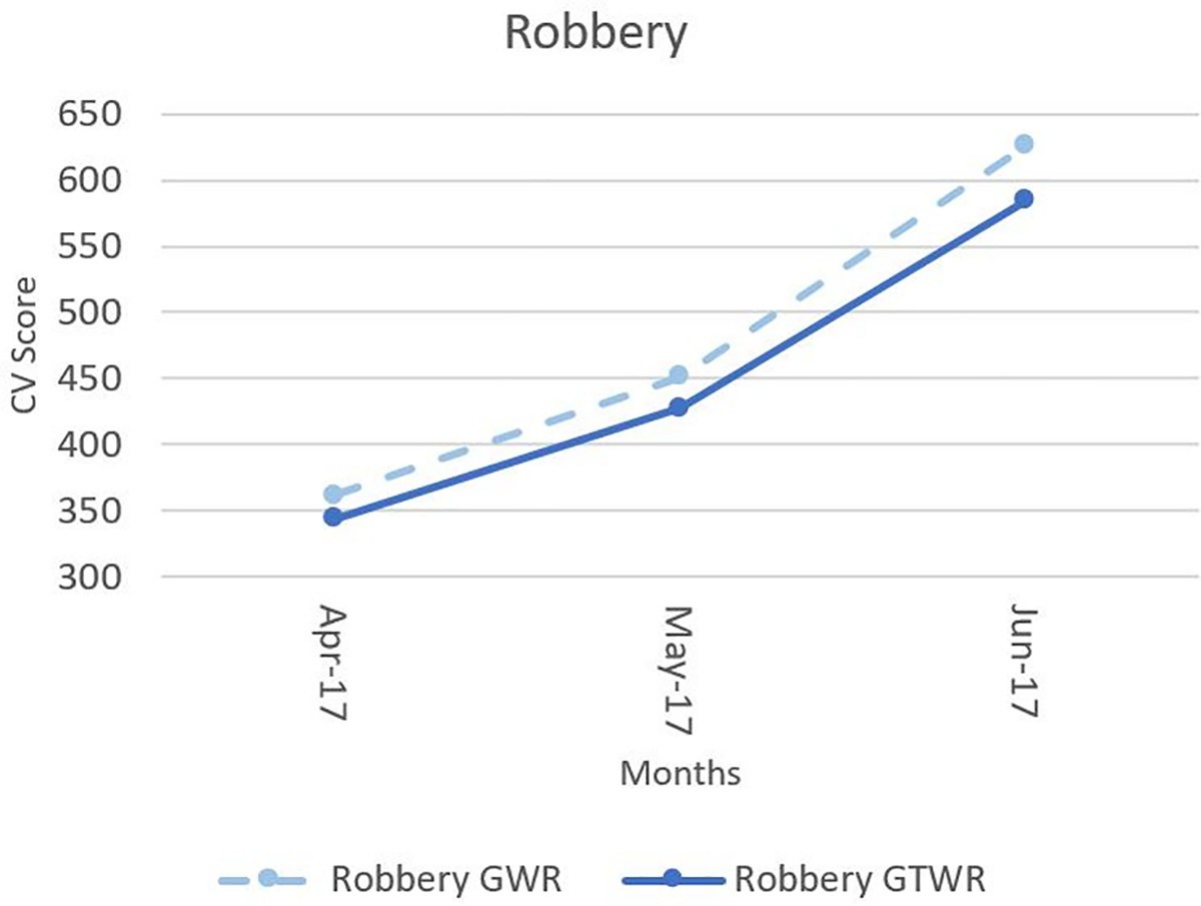
Detail illustration of cross-validation score from the GTWR and cross-sectional GWR results of robbery.

In addition, Fig 5 provides more detail zoom-in comparison of GWR and GTWR models for robbery (based on Fig 4 box part from April to June), it becomes clear that the CV score of the GTWR model is lower than the GWR model which illustrates that the performance of the GTWR model is better than the GWR model.

### 3.3 The spatial-temporal distribution of crime

Table 6 shows the model performance and the corresponing coefficients (OLS, median of GWR and GTWR). The socio-economic variables such as female ratio, and higher level of education were shown to have varied relations with robbery, domestic violence, and fraud. First, a higher female ratio had a significant positive association with domestic violence and a negative correlation to robbery. According to Miller and McCaw [60], most victims of domestic violence are women; therefore, a location with a higher female ratio is likely to have more domestic violence cases. In addition, areas with low education populations tend to have more domestic violence. Based on Hoffman et al’s, [61] findings, when the husband cannot fulfill his role as provider due to his low education level, he may feel threatened and become violent to his wife and children in an attempt to exhibit his “authority.” In contrast, likely targets of robbery are employed, physically weak or drunk men due to easy access to money for the thieves. However, fraud victims are usually chosen randomly. Higher education levels lead to occupational advantages; however, rich people are often victims of robbery and fraud.

Population, divorce rate, and unemployment rates were significantly positively correlated with domestic violence, robbery, and fraud, while the median age was significantly negatively correlated with these crime. Despite the fact that the divorce rate had the same association compared to other crimes, it did not have a significant association on robbery. In terms of weather conditions, the temperature has been found to have a significant positive correlation with domestic violence which is consistent with Shen et al, [62] findings. On the other hand, Rainfall had a positive association with fraud and robbery and negative relations with domestic violence. In this case, it is contrary to the hypothesis that extreme rainfall would have a positive association with domestic violence because it is an indoor crime. Instances of robbery, which is an outdoor crime, would be down because fewer people would be outside. However, according to our model, the correlation between rainfall was insignificant except to fraud.

According to temporal analysis in the GTWR model, there was no significant coefficient changes in socio-economic variables throughout 2016 till 2018 (because no significant changes in most socio-economic values from month to month).

**Fig 6:**
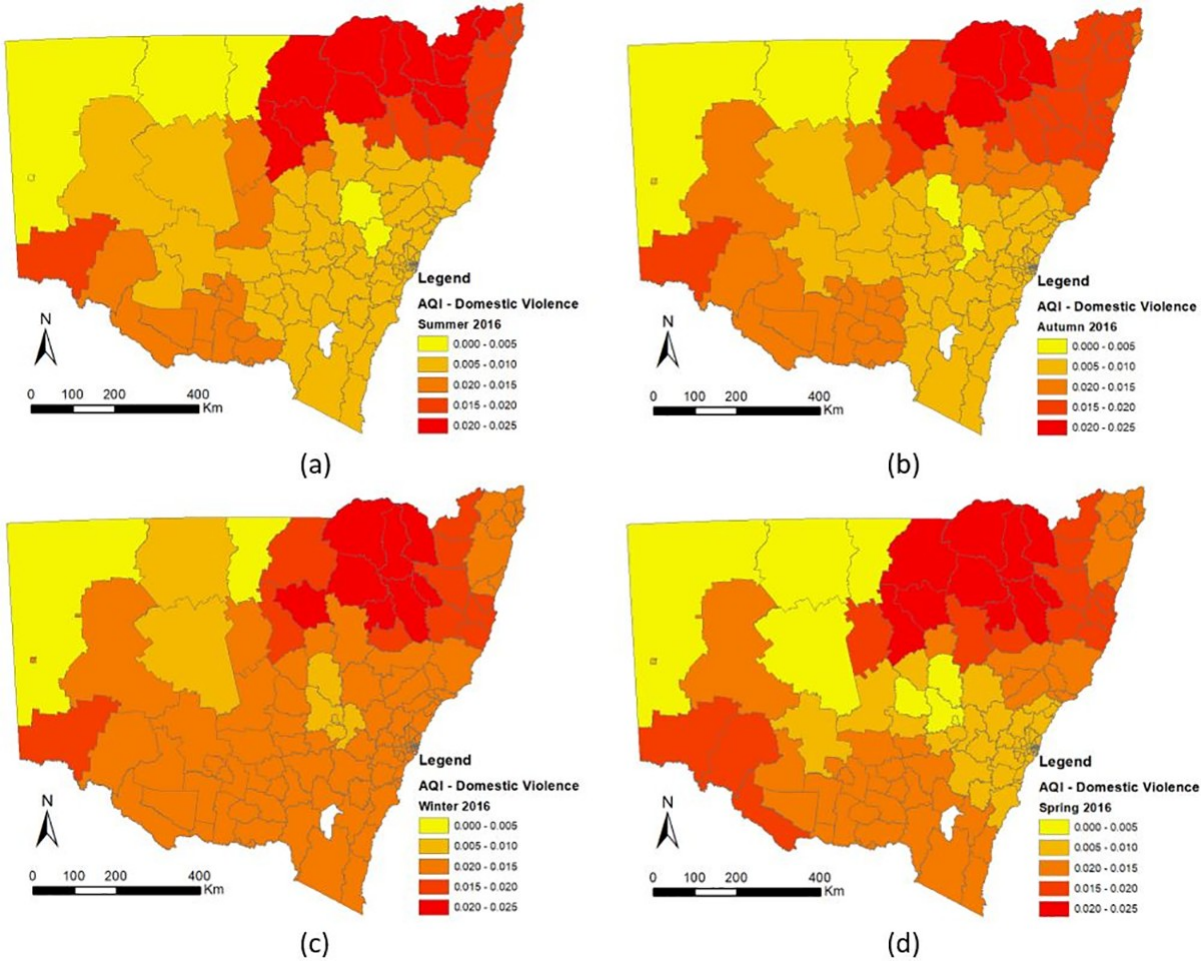
Spatial-Temporal Difference of AQI Coefficients of Domestic Violence in New South Wales (a) Summer 2016, (b) Autumn 2016, (c) Winter 2016, (d) Spring 2016.

**Fig 7:**
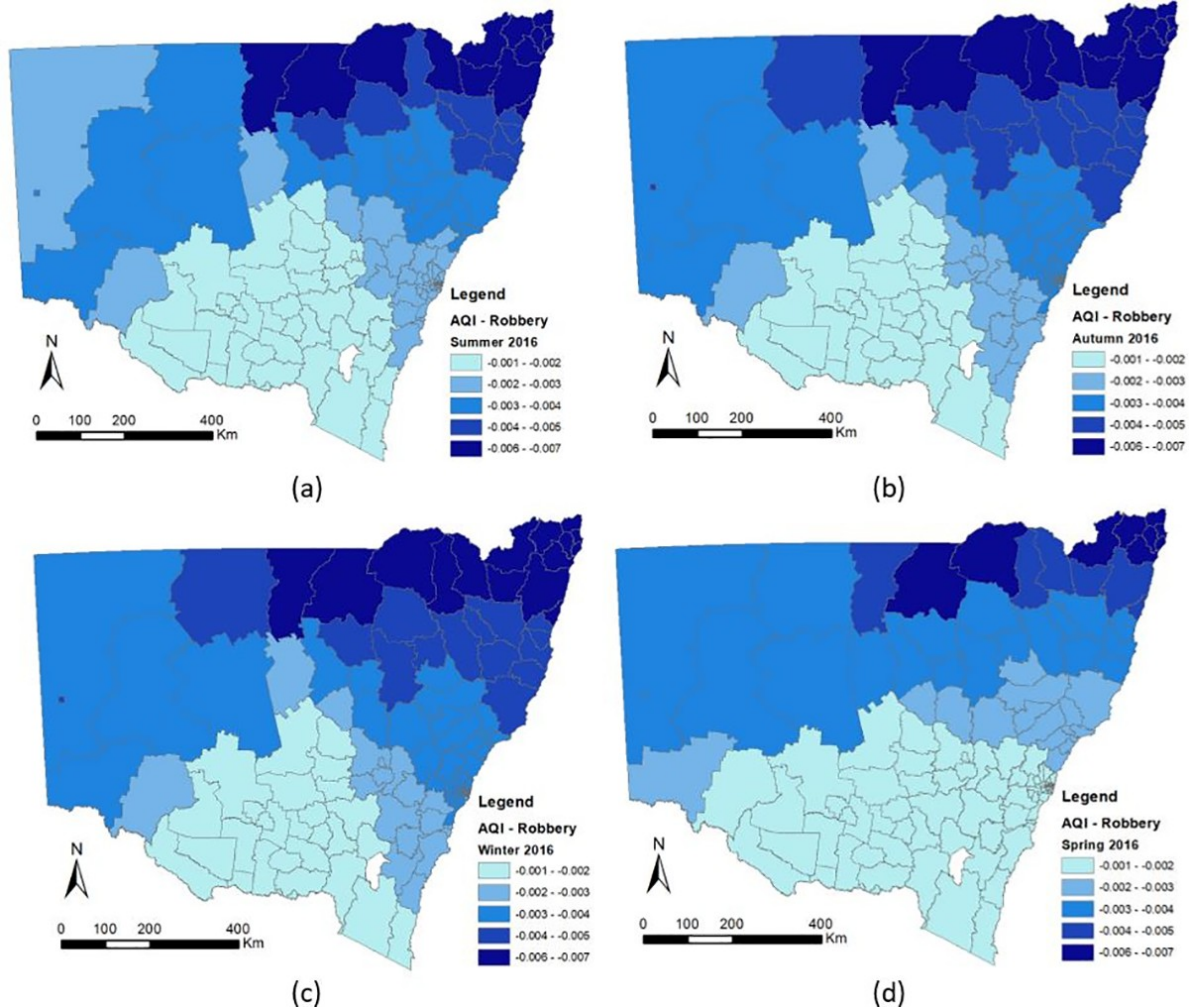
Spatial-Temporal Difference of the AQI Coefficients of Robbery in New South Wales (a) Summer 2016, (b) Autumn 2016, (c) Winter 2016, (d) Spring 2016.

**Fig 8:**
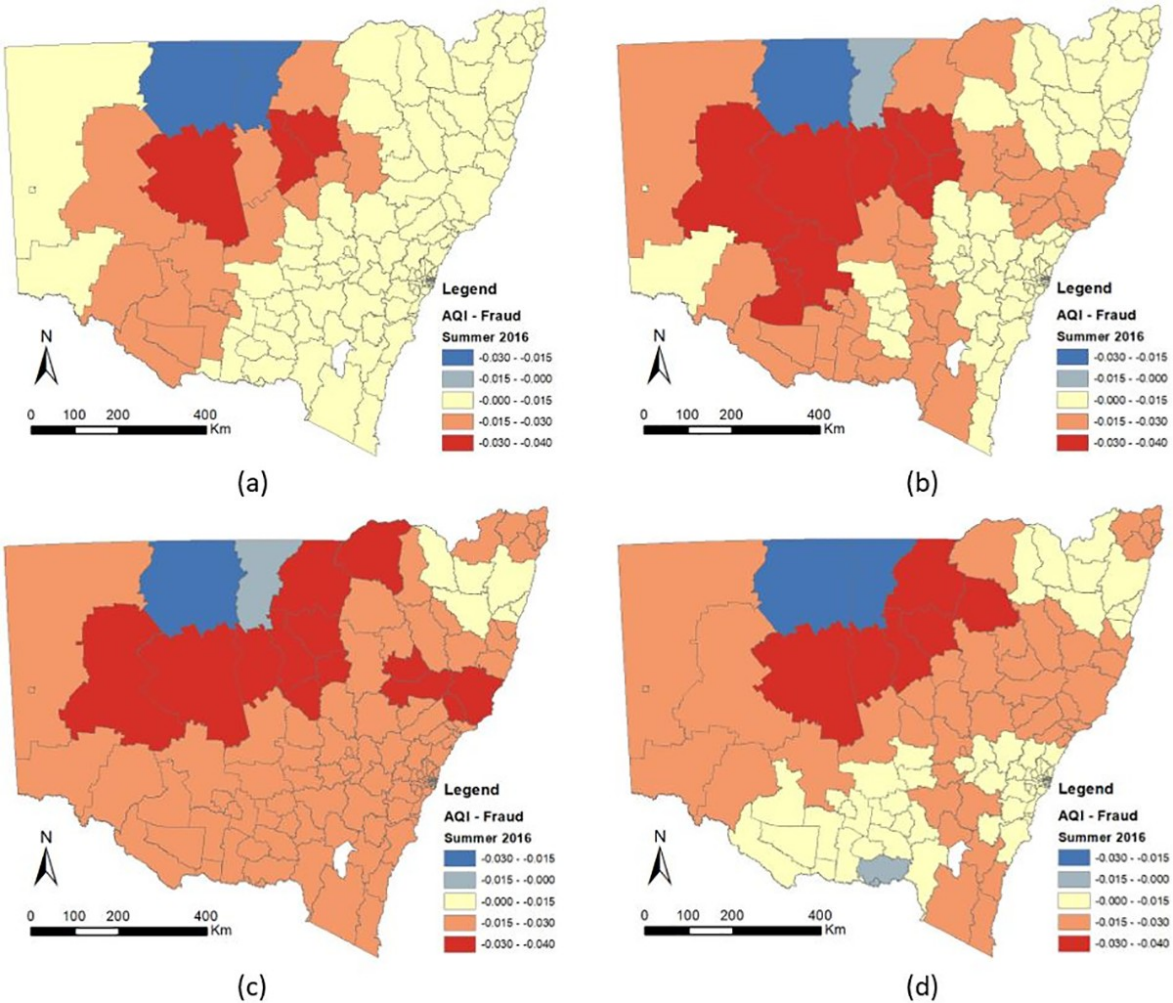
Spatial-Temporal Differences of AQI Coefficients of Fraud in New South Wales (a) Summer 2016, (b) Autumn 2016, (c) Winter 2016, (d) Spring 2016.

The following discussion will focus on the association between air quality and crime. Detailed information about the spatial-temporal changes of the coefficients of AQI were shown in Figs 6–8, which illustrate the estimated coefficient variation of AQI in the GTWR for each crime. Cold colors (blue) represent negative coefficient values, while warm colors (red) represent positive ones. Specifically, according to the GTWR results, the coefficient correlation between air pollution and all types of crime (domestic violence, robbery, fraud) continued to increase from the beginning (Summer) to the end of the year (Spring) and the spatial effect increased from rural to urban areas.

#### 3.3.1 Domestic violence

Fig 6 illustrates a positive significant association between high pollution levels and domestic violence because all AQI coefficients are positive. In other words, the higher the AQI value in the area, the higher the rates of domestic violence. According to the local model results, there was a difference in every AQI coefficient between the locations and seasons. The AQI coefficient values decreased from urban to rural (from east to the west). This shows that when air pollution becomes worse, rates of domestic violence increase more significantly in urban areas as compared to rural regions. Domestic violence rates also fluctuate according to the seasons. As air pollution becomes more serious during the winter than summer, the coefficients of AQI in the winter are higher than those in the summer for domestic violence. Moreover, this result is consistent over the three-year study period.

#### 3.3.2 Robbery

Similarly, Fig 7 show the changes in the coefficients of the AQI variable for the GTWR model with regard to robbery. However, high levels of air pollution were found to have the opposite correlation with domestic violence. The AQI had a significantly negative association with robbery. The mean coefficient value of the AQI was -0.024 for the GTWR model, and the minimum and maximum estimates were both negative. Also, the distribution range was small (-0.05 - -0.015 for the GTWR). The results show that severe air pollution decreases the number of robberies, especially in urban areas. Temporally, because of the negative correlation between AQI and robbery, the coefficient value of AQI becomes increasingly negative from summer to winter. Based on our three-year dataset, the level of air pollution during winter was found to be more serious than in summer and the seasonal coefficient distribution on 2017, 2018 are similar as those on 2016. This means that the cases of robbery escalated in the summer.

#### 3.3.3 Fraud

Similar to domestic violence, the AQI coefficient was significantly positively associated with fraud. As shown in Fig 8, the results indicate that areas with poor air quality are more likely to experience more cases of fraud. From the spatial distribution of the AQI coefficient, it is clear that urban areas have higher correlation with air pollution than rural regions. The distribution range was -0.03 to 0.04 for GTWR. In the GTWR model, 98.7% of the coefficients were positive, with 0.012 mean values for the AQI coefficient. With regard to seasonal effects, it became clear that fraud occurred most frequently in winter and lessened in summer over the three-year study period.

## 4 Discussion and conclusions

### 4.1 Discussion

In this study, the association between air pollution and crime were examined by using spatial-temporal models. The GTWR model results showed that air pollution has an inconsistent correlation with various types of crime. The areas with higher levels of air pollution are likely to have more domestic violence cases (indoor crime) but fewer incidences of robbery (outdoor violence) because it changes people’s daily activities by keeping them inside for longer periods of time. According to Oltra and Sala [63], people in different regions have different perceptions of the risk caused by air pollution that could influence their decisions about going outdoors. For example, people who live in the West, such as Europeans, are frequently affected by air pollution. In other words, people who live in areas with better air quality will have a more positive perception of their home environment [64]. In order to avoid exposure to air pollution, they choose to stay at home on days with high air pollution [65]. On the other hand, residents of countries with an exceptional scale of industries that cause pollution (especially low-income people) have less perceived risk with regard to being exposed to air pollution [66] since they prioritize to pursuit a better economic growth; however, many of them still cancel their outdoor activities when warned of extremely poor air quality [67,68]. Based on geotagged social media check-in analysis conducted by Yan et al. [69], an increase in air pollution will reduce overall urban activities, such as leisure and work-related pursuits, to decrease the risk of exposure. Lastly, fraud may occur randomly in both public and private places. High levels of air pollution have been shown to increase fraud. This may be due to the impacts of air pollution on mental health, such as stress and depression.

In addition, this study includes a detailed discussion of each type of crime. First, air pollution was found to have a positive and significant association with domestic violence, meaning that areas with more serve air pollution (higher AQI) tend to have more cases of domestic violence. This finding is consistent with previous studies that shows a higher air pollution level has a significant positive correlation with violent crime [12,40,41]. Besides, according to quasi-experimental from Herrnstadt and Muehlegger [10,38], the authors found the neighbourhood on the downwind side experienced an increase in the incidence of violent crime due to strong wind that brought excessive concentration air pollution. The result from [11,41,70] revealed that exposure to air pollution could alter the impairment of cognition, including anxiety, depression, and aggression to human. Studies undertaken in Asia also did yield an association between short-term air pollution exposure and suicide risk [71], depressive symptoms [72], and emergency department visits for depression [73]. Furthermore, exposure to common air pollutants may impact a much wider set of economic and social outcomes. Therefore, due to the influence of various factors, short-term exposure to pollution can change the way people feel and behave, such as making people more irritable [39]. This study assumes this effect is more detrimental for people who stay in couples. Potentially abusive males who already exposed to air pollution may be under a state of anxiety and tend to abuse their partners (especially women). This may explain the positive relationship between air pollution and domestic violence. However, since this study used monthly crime data, we could not conduct a quasi-experiment in order to observe the direct impact of air pollution on domestic violence. Nevertheless, our result able to illustrates how the correlation of which increases from rural to urban areas (Sydney, Wollongong, Newcastle, and Albury). The overall results of this study were as expected and consistent with current research [6,25,74]. The variables detailed above could lead to domestic violence, an indoor crime. Moreover, air pollution also could intensify mental illness that may lead to violent behavior.

Furthermore, the coefficients of AQI were negatively associated with robbery, which means there are fewer robberies in areas with severe pollution. This result was consistent with the assumption that people tend to stay at home and postpone or reschedule their outdoor activities to avoid possible health problems associated with air pollution [69]. This situation will discourage potential criminals simply because they would have fewer targets outside. Moreover, high levels of air pollution could also reduce property crimes because people can better protect their belongings when they are at home. Therefore, population density has a positive and statistically significant relationship with this crime. This result is consistent with the findings of Boivin [75] who determined that areas with large populations, whether permanent, tourists or a combination of both, would be expected to experience more crime because a larger population provides more potential targets and offenders.

Finally, the coefficient of AQI was found to be somewhat less significantly related to fraud compared to the other types of crimes. According to fraud records, these crimes are highly correlated with a person’s lack of morality, supervision, and knowledge about this crime as well as high levels of peer pressure [76]. Lack of morality and stress could also come from the effects of air pollution that indirectly leads people **to** commit this act. Most fraud victims are people who want to attain a certain lifestyle. In general rule, the higher the person’s level of education, the greater the earning potential [77]. With the limitation of salary, the lower educational background may have difficulties to fulfill their lifestyle that may lead to conduct a fraud. Therefore, an education degree and unemployment rate is a significant factor that is suitable for this study result.

With regard to the relation of AQI to crime, the difference between its temporal-spatial distribution is obvious (Figs 6–8) and the association between air pollution and all types of crime have continued to increase from the beginning to the end of the year (summer to winter). This occurs because the AQI changes every month or even every day. Researchers have already determined that the AQI value is significantly affected by the season. In addition concentration of air pollution are associated with temperature and wind speed, according to Czarnecka and Nidzgorska-Lencewicz [78]. Therefore, the number of crimes could change seasonally, whereas if the AQI values change, the number of crimes could either increase or decrease depending on the type of crime.

### 4.2 Conclusions

In this paper, the spatial-temporal models were compared to other traditional models to determine if air pollution had an association with various types of crime. Three types of crime were highlighted in this study namely: domestic violence (indoor crime), robbery (outdoor crime), and fraud (an indoor cyber crime). Based on the spatial and temporal stationarity test results, all variables exhibited extra variation, which means the GTWR model is the most effective in this case. In addition, the GTWR also performed better with the highest R-square and AIC value compared to other models. It shows that air pollution and other related factors have very different spatial and temporal coefficient distributions regarding these three types of crimes. Overall, according to the model, air pollution was found to be positively related to indoor and cybercrimes (domestic violence and fraud). However, air pollution was also found to have a negative association with outdoor crimes (robbery). Moreover, its relation was spatially heterogeneous.

On the other hand, this study also has several limitations. First, the census data used in this study may not be the "best" community-level unit. Smaller census block groups may be preferred. However, the smaller units in this study area are not suitable because the number of robbery and domestic violence counts is too low and count distributions with too many zeros. Second, the temporal resolution used in this study is still in a period of months that can only provide a rough illustration between the association of air pollution and crime. Although monthly averages are useful for district commanders in developing seasonal crime-fighting strategies, different patterns on a smaller time scale can emerge. On the contrary, the concentration of air pollution also changes every time starting from day to hour. Here, attempts to use smaller temporal units are not feasible due to limitations access to the data from the government. Third, due to the limitation of traffic count in rural areas of New South Wales, this study could not further observe the impact of air pollution on outdoor activities.

In the future, air pollution is expected to worsen due to climate change. For this reason, future researchers should continue to study the relationship between air pollution and domestic violence. Long-term law enforcement to reduce crime must be developed. Specifically, greater efforts need to be made in the prevention of comprehensive domestic violence by considering environmental factors. Therefore, local government officials should consider the relation between air pollution and their domestic violence prevention strategies. Although monthly data was used in this study to determine the association between air pollution and crime, future scholars will include daily data to more clearly illustrate the correlation between air pollution and various types of crime.

## Appendix A

**Table:** Correlation matrix of variables.

	Domestic Violence	Robbery	Fraud	Population	Female Ratio	Median Age	Income	Education	Unemployment	Divorce Rate	Min Temperature	Mean Rainfall	AQI
Domestic Violence	1.00	0.45	0.78	0.87	0.41	-0.40	-0.06	-0.06	0.69	-0.05	0.17	0.09	0.33
Robbery		1.00	0.55	0.46	0.14	-0.26	-0.01	0.08	0.39	-0.05	0.00	0.14	-0.15
Fraud			1.00	0.82	0.25	-0.46	0.14	0.24	0.49	-0.12	0.17	0.13	0.32
Population				1.00	0.43	-0.45	0.17	0.17	0.61	-0.12	0.21	0.16	0.34
Female Ratio					1.00	-0.11	0.13	0.16	0.48	0.06	0.14	0.14	0.17
Median Age						1.00	-0.31	-0.31	-0.28	0.36	-0.11	-0.06	-0.20
Income							1.00	0.72	-0.19	-0.23	0.11	0.10	0.25
Education								1.00	-0.14	-0.24	0.13	0.13	0.24
Unemployment									1.00	0.04	0.13	0.07	0.10
Divorce Rate										1.00	-0.02	0.00	-0.08
Min Temperature											1.00	-0.13	0.27
Mean Rainfall												1.00	-0.10
AQI													1.00
